# Response to the “Letter to the Editor” by Alfred Körblein, “Short term increase in low birthweight babies after Fukushima”

**DOI:** 10.1186/s12940-020-00675-x

**Published:** 2020-11-25

**Authors:** Hagen Scherb, Keiji Hayashi

**Affiliations:** 1 Helmholtz Zentrum München, German Research Center for Environmental Health, Institute of Computational Biology, Ingolstädter Landstr. 1, 85764 Neuherberg, Germany; 2 Hayashi Children’s Clinic, 4-6-11-1F Nagata, Joto-ku Osaka-Shi, Osaka, 536-0022 Japan

**Keywords:** Radiation-induced genetic effects, Scientific logic, Statistical modeling, Statistical inference

Dear Editors,

We wish to thank Alfred Körblein for raising methodological and practical issues as to how to adequately assess possible changes in the trend/s of low birth weight proportions in Japan before and after the Fukushima Daiichi Nuclear Power Plant (FDNPP) accidents [1]. Alfred Körblein’s letter provides an opportunity to explaining in detail several technical and crucial aspects of our approach to data analysis [2].

In his Fig. 1 (upper panel), Körblein fits a 5th degree polynomial logistic regression model to the combined low birth weight data of the five moderately and five highly contaminated prefectures Chiba, Fukushima, Ibaraki, Iwate, Kanagawa, Miyagi, Saitama, Tochigi, Tokyo, and Yamagata. Körblein reports a jump in this trend in 2012 with an odds ratio (OR) of 1.019, 95%-confidence interval (0.994, 1.044), *p*-value 0.152, which we confirm in principle. However, whereas Körblein employs the t-distribution for computing *p*-values in this example with 24 data points, 7 parameters (intercept, jump_2012_, t = time, t^2^, t^3^, t^4^, t^5^), and 17 degrees of freedom, we consider the Wald-Chi^2^ a more appropriate and less conservative choice. The Wald-Chi^2^ (with optional adjustment for overdispersion) is the default distribution of logistic regression in SAS.

**Fig. 1:**
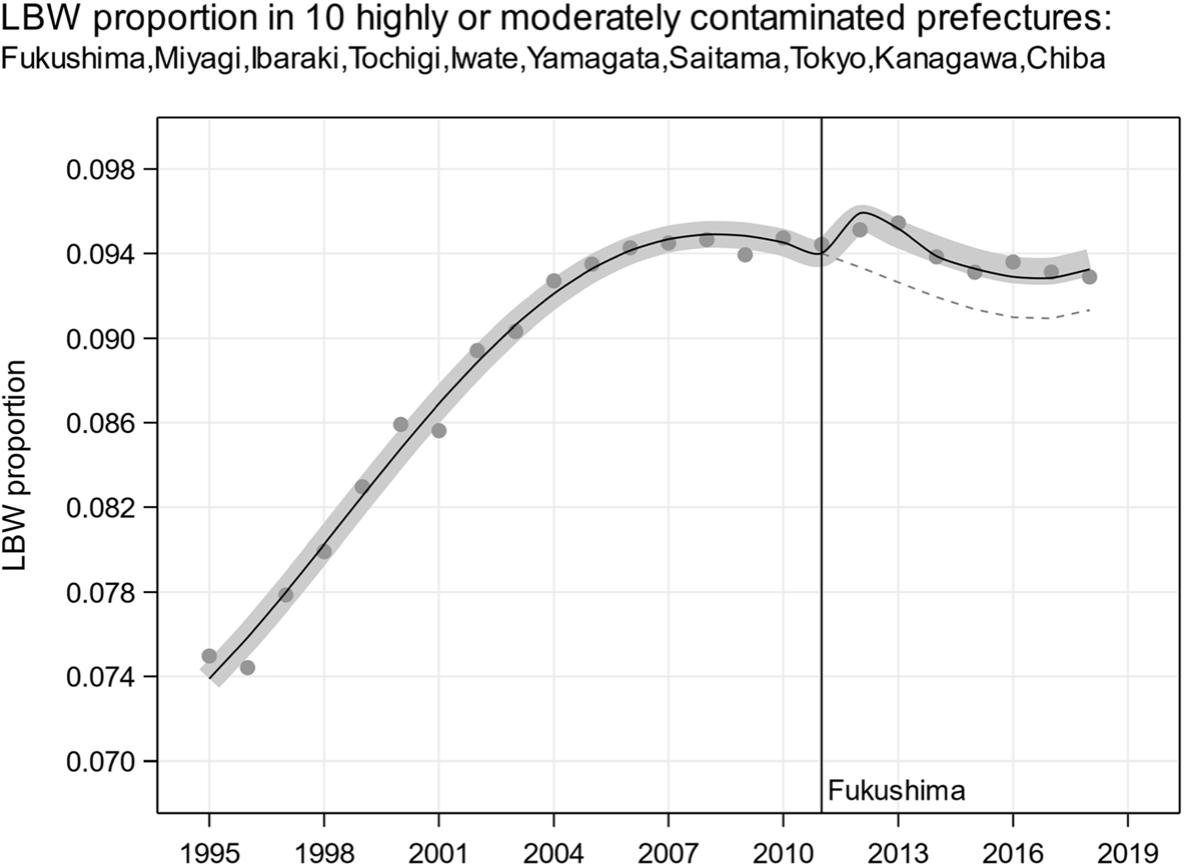
Low birth weight (LBW) proportion in 10 moderately or highly contaminated prefectures Chiba, Fukushima, Ibaraki, Iwate, Kanagawa, Miyagi, Saitama, Tochigi, Tokyo, and Yamagata 1995 to 2018; 4th degree polynomial logistic regression trends allowing for jumps from 2012 onward; thick gray line: jump 2012 to 2018 OR 1.027, (1.004, 1.051), *p*-value 0.0203; thin black line: jump 2012 to 2013 OR 1.027, (1.003, 1.052), p-value 0.0244 and jump 2014 to 2018 OR 1.024, (0.991, 1.059), p-value 0.1547

Körblein’s approach is motivated by the truism that a 5th degree polynomial fits the data better than a 4th degree polynomial. However, fit in terms of deviance is not the only important component in this context. If the polynomial degree is increased, variance inflation, over-fitting, and over-adjustment may become problematic. In order to illustrate this, consider the extreme case of a 23rd degree polynomial for the 24 data points in our examples. Such a polynomial would theoretically pass through all given data points, but it would not be possible to compare or mutually test segments of the regression line [3]. Principles that should guide the selection of an appropriate degree of the polynomial include parsimony and the precision of the regression coefficients. The Wald Chi^2^ *p*-values for t^4^ and t^5^ of the 5th degree polynomial are 0.0538 and 0.1331, respectively. By contrast, p for t^4^ in a 4th degree polynomial is only 0.0003, i.e. a more parsimonious polynomial yields a more precise estimate of t^4^. These considerations apply in principle to all four scenarios in Fig. 4 of our paper [2] as per Table 1. Since 4th degree polynomials are more parsimonious and yield more precise estimates (due to lesser variance inflation) of the regression coefficients when compared to 5th degree polynomials, we recommend the use of 4th degree polynomials in this context.

**Table 1:** *P*-values for t^4^ versus t^4^ and t^5^ of the 4th and the 5th degree polynomial logistic trend models, respectively; A: Japan; B: Japan excluding 10 exposed prefectures; C: 5 moderately exposed prefectures (Yamagata, Saitama, Tokyo, Kanagawa, Chiba); D: 5 highly exposed prefectures (Fukushima, Miyagi, Ibaraki, Tochigi, Iwate)

Scenario	Variable	*p*-values (Wald Chi^2^)	
		4th degree polynomial	5th degree polynomial
**A**	t^4^	< 0.0001	0.0080
	t^5^	./.	0.0503
**B**	t^4^	0.0004	0.0337
	t^5^	./.	0.0882
**C**	t^4^	< 0.0001	0.0568
	t^5^	./.	0.1586
**D**	t^4^	0.0926	0.1616
	t^5^	./.	0.2255
**C + D**	t^4^	0.0003	0.0538
	t^5^	./.	0.1331

Körblein’s Fig. 1 (lower panel) is scientifically unsound. Since the 5th degree polynomial provides a superior fit and the estimated jump in 2012 is ‘insignificant’ (*p*-value > 0.05), Körblein tests a jump restricted to the years 2012 and 2013. This approach assumes that the environmental exposure situation after 2013 is the same as before the FDNPP accidents. We consider Körblein’s amalgamating the periods 1985 to 2011 and 2014 to 2018 in order to obtain a baseline trend illogical since the FDNPP accidents released long-lived radioactive elements. This approach also ignores that radiological accidents have been followed by long-term radiation-induced genetic effects [4–14]. Using a 4th degree polynomial in place of a 5th degree polynomial for modeling the low birth weight proportion in the 10 moderately or highly contaminated prefectures reveals a significant jump in 2012 with OR 1.027, (1.004, 1.051), *p*-value 0.0203, see Fig. 1. The division of the period 2012 to 2018 into two periods, 2012 to 2013 and 2014 to 2018 yields a somewhat weaker and less precisely estimated effect in the second period compared to the former: OR 1.024, (0.991, 1.059), p-value 0.1547. This reduced effect in a later period is compatible with the decrease in exposure due to radioactive decay and decontamination [13].

While Körblein’s statement ‘*the significant result for the shift in LBW proportion obtained with model 1 is driven by the peak in 2012-2013’* is true in several selected scenarios within his framework, the *p*-value of > 0.05 for the jump from 2014 onward in Fig. 1 is certainly not evidence of absence of long-term genetic effects. This type of erroneous interpretation of *p*-values has frequently raised criticism in the past. A more recent critique has been published in *Nature:* ‘*Let’s be clear about what must stop: we should never conclude there is ‘no difference’ or ‘no association’ just because a P value is larger than a threshold such as 0.05. Neither should we conclude that two studies conflict because one had a statistically significant result and the other did not. These errors waste research efforts and misinform policy decisions*’ [15].

In summary, Körblein’s conclusions hereunder evolve from misinterpreted analysis:

- ‘*An analysis of low birth weight (LBW) births in ten contaminated prefectures of Japan, 1995-2018, finds a statistically significant increase in the LBW proportion in 2012-2013, but no increase after 2013.*’
- ‘*The claim by Scherb that their result is evidence of a genetic radiation effect is challenged by the present analysis.*’

Sincerely,

Hagen Scherb and Keiji Hayashi

## Data Availability

The employed data has exclusively been published previously and/or it is contained in the Tables and in the Figures included in this paper.

## Abbreviations

95%-CI or (.,.): 95%-confidence interval
FDNPP: Fukushima Daiichi Nuclear Power Plant
LBW: Low birth weight
OR: Odds ratio
p: p-value
SAS: Statistical Analysis System, software produced by SAS Institute Inc
